# Dexmedetomidine protects cardiomyocytes against hypoxia/reoxygenation injury via multiple mechanisms

**DOI:** 10.1002/jcla.24119

**Published:** 2021-12-09

**Authors:** Shunv Cai, Yixing Liu, Yun Cheng, Junbo Yuan, Jun Fang

**Affiliations:** ^1^ Department of Anesthesiology The Cancer Hospital of the University of Chinese Academy of Sciences (Zhejiang Cancer Hospital) Hangzhou Zhejiang China

**Keywords:** apoptosis, dexmedetomidine (Dex), ischemia/reperfusion (I/R) injury, myocardial infarction (MI), oxidative stress

## Abstract

**Background:**

Myocardial infarction (MI) is a serious cardiovascular disease associated with myocardial ischemia/reperfusion (I/R) injury. Dexmedetomidine (Dex), an α2‐adrenoceptor agonist, has been reported to protect against I/R injury. We examined the cardioprotective effects of Dex on cardiomyocytes under hypoxia/reoxygenation (H/R) conditions and explored the underlying mechanisms.

**Materials and methods:**

A H/R model was established to mimic the MI injury. The CCK‐8 assay was performed to measure cell viability. Cellular apoptosis was measured using the Annexin V fluorescein isothiocyanate (FITC)‐propidium iodide (PI) staining. The levels of interleukin (IL)‐1α and tumor necrosis factor (TNF)‐α, and the activity of lactate dehydrogenase (LDH) were measured using a commercial enzyme‐linked immunosorbent assay (ELISA) kit. Reactive oxygen species (ROS) were measured using the 2'‐7’ dichlorofluorescein diacetate (DCFH‐DA) staining assay. In addition, the levels of malondialdehyde (MDA) and the activity of superoxide dismutase (SOD), catalase (CAT), and caspase‐3 were measured using a commercial kit. siRNA was used to silence Bcl‐2, catalase, or STAT3. Western blotting was used to measure the change in the levels of proteins.

**Results:**

Dex improved the cell viability and inhibited the inflammatory response in H9c2 cells exposed to H/R treatment. In addition, Dex inhibited apoptosis and alleviated the endoplasmic reticulum (ER) stress and oxidative stress in H9c2 cells under the H/R treatment. Mechanism investigation showed that Dex inhibited the intrinsic pathway of apoptosis. Moreover, Dex enhanced the activation of the JAK2/STAT3 signaling pathway in H/R‐treated H9c2 cells.

**Conclusion:**

Altogether, our findings suggested Dex as a promising therapeutic agent for myocardial I/R.

## Graphical abstract

Dexmedetomidine (Dex) is an α‐2‐adrenoceptor agonist. In the present study, we revealed that Dex is able to increase cell viability and inhibited the inflammatory response in H9c2 cells under the hypoxia/reoxygenation (H/R) treatment. Mechanistically, Dex treatment led to the activation of JAK2/STAT3 pathway which is responsible for the inhibition of the intrinsic apoptotic pathway, endoplasmic reticulum stress, and oxidative stress. Thus, Dex might be a promising agent for the treatment of myocardial I/R.

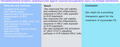

## 1 INTRODUCTION

Myocardial infarction (MI) is one of the leading causes of death worldwide.^1^ Oxidative stress, triggered by an imbalance between the accumulation of reactive oxygen species (ROS) and the efficiency of the antioxidant system to remove ROS, can cause irreversible damage to cellular components.^2^ These irreversible changes consequently trigger cellular mortality, cell death, and finally MI.^3^ In addition, excessive generation of ROS could trigger endoplasmic reticulum (ER) stress that has been implicated in myocardial I/R injury.^4^ Hence, targeting oxidative stress could be a promising strategy for the treatment of MI.

Dexmedetomidine (Dex), a highly selective α2‐adrenoceptor agonist with cardioprotective activity,^5^ possesses several other biological functions such as neuroprotective, lung protective, and renal protective effects against I/R injury.^6^ Dex is known to repress oxidative stress and inflammatory response by inhibiting the sympathetic excitability caused by α‐2 receptor stimulation.^7^ Although Dex provides protective effects against I/R‐induced injury in various cells including cardiomyocytes, its function in myocardial I/R injury is not completely understood. In the present study, we investigated the protective effects of Dex against apoptosis during I/R injury and the underlying mechanisms. Our results suggested that Dex protects against I/R injury by inhibiting intrinsic apoptosis and ER stress via the activation of the JAK/STAT3 signaling pathway. Our findings suggest that Dex could serve as a potential therapeutic agent for MI.

## 2 MATERIALS AND METHODS

### 2.1 Establishment of H/R model

H9c2 cells were cultured under hypoxic conditions of 95% N_2_ and 5%CO_2_ for 4 h at 37°C. Next, the medium was replaced with a fresh oxygenated culture medium in a normoxic incubator for 6 h at 37°C. The cells under normoxic conditions were used as a control.

### 2.2 Cell viability assay

Cell viability was measured using the CCK‐8 assay kit (Beyotime) according to the manufacturer's protocol. Briefly, H9c2 cells were seeded at a density of 5 × 10^3^ cells/well in 96‐well plates and pretreated with different doses of Dex for 24 h. Next, the cells were cultured under the H/R conditions for 4 h, and the CCK‐8 reagent (10 μl) was added to each well and cultured for another 3 h at 37°C. The absorbance at 590 nm was read using a microplate reader (BioTek).

### 2.3 Measurement of apoptosis

Cellular apoptosis was measured using the Annexin V fluorescein isothiocyanate (FITC)‐propidium iodide (PI) staining kit (Sigma) according to the manufacturer's protocol. Briefly, the cells were collected after different treatments and washed thrice with cold phosphate‐buffered saline (PBS) by centrifugation for 5 min at 500 g and re‐suspended at a density of 1 × 10^6^/ml. Cells (500 μl) were next stained with the Annexin V FITC (5 μl) and PI (10 μl) and placed in the dark for 15 min. The results were analyzed by flow cytometry (FACS Calibur^TM^, BD Biosciences). The data of fluorescence intensity were analyzed using the FlowJo software. Each experiment was repeated at least four times.

### 2.4 Measurement of ROS

The cellular ROS was determined using a ROS assay kit (Abcam) according to the manufacturer's protocol. Briefly, after different treatments, cells were washed with PBS and incubated with 10 μM 2'‐7'dichlorofluorescin diacetate (DCFH)‐DA at 37°C for 0.5 h in the dark. Next, cells were analyzed by flow cytometry (BD Biosciences). Each experiment was repeated at least four times.

### 2.5 ELISA

The levels of interleukin (IL)‐1α, tumor necrosis factor (TNF)‐α, and lactate dehydrogenase (LDH) were measured using an IL‐1 α ELISA Kit (Abcam), TNF‐α ELISA Kit (Abcam), and LDH ELSA kit (Abcam), respectively, according to the manufacturer's protocol.

### 2.6 Measurement of MDA, SOD, CAT, and caspase‐3 activities

The levels of malondialdehyde (MDA), and activities of superoxide dismutase (SOD), catalase (CAT), and caspase‐3 were measured using the MDA assay kit (Abcam), SOD assay kit (Abcam), catalase assay kit (Abcam), and caspase‐3 assay kit (Abcam), respectively, according to the manufacturer's protocol.

5′‐GGA TGC CTT TGT GGA ACT GTA TT‐3′ (sense) and

3′‐TAC AGT TCC ACA AAG GCA TCC‐5–5′ (antisense).

5′‐GGA TGC CTT TGT GGA ACT GTA TT‐3′ (sense) and

3′‐TAC AGT TCC ACA AAG GCA TCC‐5′ (antisense).

### 2.7 Dual‐luciferase reporter assay

Promoter constructs for the assays were generated by the Shanghai BioWon Biotechnology Ltd. The wild‐type and mutant catalase promoter regions were subcloned into the pGL3‐Basic vector. H9c2 cells were co‐transfected with catalase‐promoter‐luc (500 ng) and *Renilla* luciferase plasmid pRL‐TK (6 ng) using Lipofectamine 2000 (Life Technologies). Four hours after the transfection, cells were treated with IL‐6 (50 ng/ml) for another 24 h. Next, cells were collected and lysed, and the relative luciferase activities were assayed using the dual‐luciferase reporter assay kit (Promega) according to the manufacturer's protocol. The results obtained were normalized to *Renilla* luciferase activity and expressed relative to the activity of the untreated control group transfected with a catalase‐promoter‐Luc vector.

### 2.8 Western blotting and immunoprecipitation assay

Cells were lysed using the CHAPS buffer, and the protein concentrations were measured using the Bradford assay kit (Beyotime). An equal amount of protein (20 μg) was resolved on 10% SDS‐PAGE and transferred to polyvinylidene fluoride (PVDF) membranes. Next, the PVDF membranes were blocked with skimmed milk for 1 h at room temperature, following which the membranes were incubated with primary antibodies overnight at 4°C. Afterward, the membranes were incubated with secondary horseradish peroxidase (HRP)‐conjugated antibodies (Sigma‐Aldrich). Immunoprecipitation was performed as described previously to detect the activation of Bax.^8^ The following primary antibodies were used: Caspase‐3 (CST, USA), Bcl‐2 (CST), Smac/DIABLO (CST), Cytochrome *c* (CST), Bax (CST), Bax (6A7) (CST), GRP78 (Abcam), CHOP (Abcam), phospho‐JAK2 (Abcam), phospho‐STAT3 (Abcam), and GAPDH (Sigma‐Aldrich). Secondary antibodies were obtained from Sigma‐Aldrich.

### 2.9 Statistical analysis

All statistical analyses were performed using SPSS12.0 (IBM). Data are presented as mean ± standard error (SD). Statistical differences were determined using the unpaired Student's *t* test or one‐way analysis of variance (ANOVA) followed by post hoc Tukey's test for multiple comparisons. A *p* < 0.05 was considered significant. All experiments were repeated at least thrice.

## 3 RESULTS

### 3.1 Dex improves cell viability and inhibits the inflammatory response of H9c2 cells after H/R treatment

**FIGURE 1:**
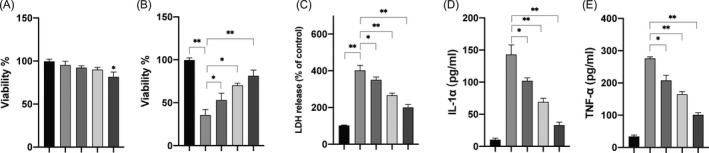
Dex ameliorated H/R‐induced injury in H9c2 cells. (A) H9c2 cells were treated with different doses of Dex for 24 h, and cell viabilities were measured. H9c2 cells were pretreated with different doses of Dex for 2 h, following which the cells were subjected to H/R treatment. (B) Cellular viabilities were assayed. (C) The release of LDH was measured. (D) Levels of IL‐1α were measured. (E) Levels of TNF‐α were measured. Data are presented as mean ± SD, *p < 0.05; **p < 0.01

H9c2 cells were treated with different doses of Dex for 24 h, and cell viabilities were measured. As indicated in Figure 1A, 40 μM Dex slightly inhibited the viability of H9c2 cells, whereas other doses of Dex (5, 10, and 20 μM) had little effect. Therefore, 5–20 μM Dex was used in the following experiments. The LDH release assay revealed that pretreatment with Dex inhibited the release of LDH after exposure to H/R (Figure 1B). Next, the effects of Dex on the viability of H/R‐treated H9c2 cells were assayed. Compared with the H/R group, the Dex‐treated groups showed markedly increased cell viability in a dose‐dependent manner (Figure 1C). In addition, secreted IL‐1α and TNF‐α were markedly blocked in the Dex‐treated group when compared with the H/R group (Figure 1D). Altogether, these data suggested that Dex mitigated the H/R injury in H9c2 cells.

### 3.2 Dex alleviates oxidative stress via the upregulation of catalase in H9c2 cells exposed to H/R treatment

**FIGURE 2:**
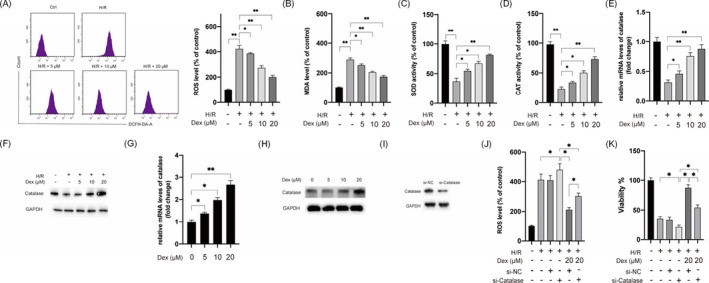
Dex alleviated H/R‐induced oxidative stress via the upregulation of catalase in H9c2 cells. H9c2 cells were pretreated with different doses of Dex for 2 h, following which cells were subjected to H/R treatment. (A) ROS levels were measured. (B) MDA levels were measured. (C) SOD activities were measured. (D) CAT activities were measured. (E) The mRNA levels of catalase were measured. (F) H9c2 cells were pretreated with different doses of Dex for 2 h, following which cells were subjected to H/R treatment and protein levels of catalase were measured by Western blotting. (G) H9c2 cells were treated with different doses of Dex for 6 h and mRNA levels of catalase were measured. (H) H9c2 cells were treated with different doses of Dex for 6 h, and protein levels of catalase were measured. (I) H9c2 cells were transfected with si‐NC or si‐catalase for 24 h, following which the protein levels of catalase were measured by Western blotting. (J) H9c2 cells were transfected with si‐NC or si‐catalase for 4 h, following which cells were treated with Dex for 2 h. Next, cells were exposed to H/R conditions, and ROS levels were measured. (K) H9c2 cells were transfected with si‐NC or si‐catalase for 4 h, following which cells were treated with Dex for 2 h, and cell viabilities were measured. Data are presented as mean ± SD, *p < 0.05; **p < 0.01

Next, we evaluated whether Dex affected the oxidative stress caused by H/R treatment. As shown in Figure 2A, the upregulation of ROS was abrogated by Dex in H9c2 cells in a dose‐dependent manner in H/R‐stimulated H9c2 cells (Figure 2A). The MDA level, SOD activity, and CAT activity were measured, which showed markedly increased MDA levels in H/R‐treated H9c2 cells compared with the control group; this enhancement was significantly inhibited by Dex pretreatment (Figure 2B). Furthermore, the pretreatment with Dex significantly enhanced the activities of both SOD and CAT in H/R‐treated H9c2 cells (Figure 2C,D). Next, the effects of Dex on the expression of antioxidant enzymes were examined. It was found that pretreatment of Dex upregulated mRNA and protein levels of catalase under H/R conditions (Figure 2E,F). Moreover, Dex treatment led to the upregulation of mRNA and protein levels of catalase under normal conditions (Figure 2G,H). However, Dex exerted little effect on the expression of MnSOD (data not shown). To validate the function of catalase in the protective effects of Dex, the catalase gene was knocked down (Figure 2I), aggravated the effects of H/R on the generation of ROS, and the viability of H9c2 cells (Figure 2J,K). The silencing of catalase abrogated the effects of Dex on the ROS levels and viability of H9c2 cells under H/R treatment (Figure 2J,K). These data suggested that Dex alleviated the oxidative stress caused by H/R treatment at least partially via upregulation of catalase activity in H9c2 cells.

### 3.3 Dex inhibits H/R‐induced apoptosis and ER stress in H9c2 cells

**FIGURE 3:**
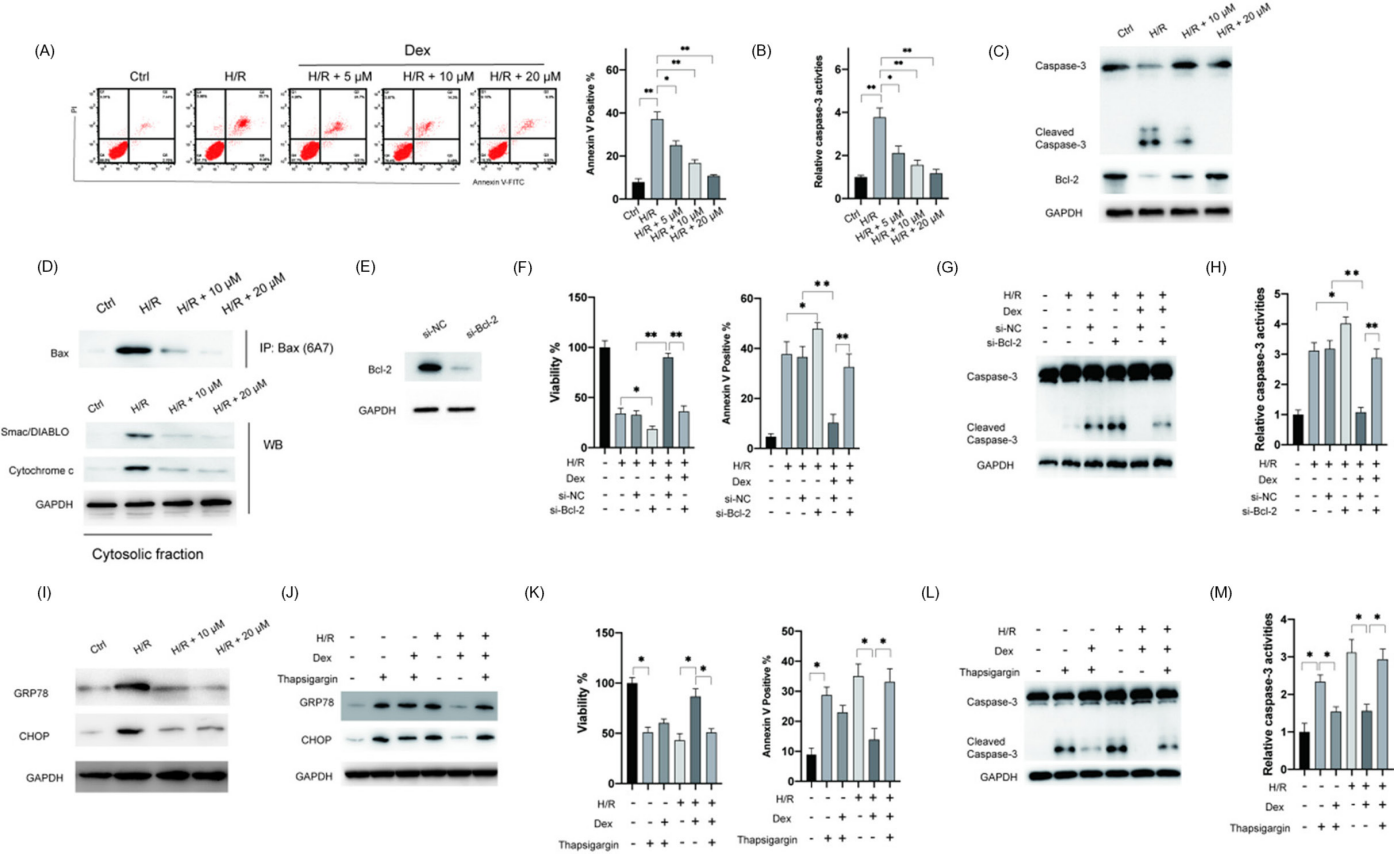
Dex inhibited apoptosis and ER stress in H/R‐treated H9c2 cells. H9c2 cells were pretreated with indicated doses of Dex for 2 h, following which cells were subjected to H/R treatment. (A) Cellular apoptosis was measured. (B) Relative caspase‐3 activities were measured. (C) Cellular lysates were subjected to Western blotting. (D) Activation of Bax was assayed by immunoprecipitation, and release of mitochondrial proteins was assayed. (E) H9c2 cells were transfected with si‐NC or si‐Bcl‐2 for 24 h, and the levels of Bcl‐2 were measured. (F) H9c2 cells were transfected with si‐NC or si‐catalase for 4 h, following which cells were treated with Dex for 2 h and then exposed to H/R condition. Afterward, cell viabilities (left) and apoptosis (right) were measured. (G) Protein levels of caspase‐3 were measured. (H) Relative caspase‐3 activities were measured. (I) Protein levels of GRP78 and CHOP were measured. (J) H9c2 cells were pretreated with Dex (20 μM) with or without thapsigargin (50 nM) for 2 h, following which cells were exposed to H/R, GRP78, and CHOP were measured by Western blotting. (K) Cell viabilities (left) and apoptosis (right) were measured. (L) Caspase‐3 levels were measured by Western blotting. (M) Relative caspase‐3 activities were measured. Data are presented as mean ± SD, *p < 0.05; **p < 0.01

Next, the effects of Dex on H/R‐induced apoptosis were examined. As shown in Figure 3A, apoptosis induced by H/R was repressed by pretreatment with Dex. Caspase‐3 activity assay and Western blotting showed that the activation of caspase‐3 was inhibited by pretreatment with Dex (Figure 3B,C). It has been documented that H/R injury induces apoptosis via the intrinsic apoptotic pathway.^9^ Therefore, we examined the effects of Dex on proteins involved in the intrinsic apoptosis and found that pretreatment with Dex successfully upregulated Bcl‐2 in H9c2 cells under the H/R condition (Figure 3C). Moreover, the pretreatment with Dex inhibited the activation of Bax and released Smac/DIABLO and cytochrome *c* into the cytosol (Figure 3D). To confirm the role of blockage of intrinsic apoptosis in mediating the protective effects of Dex, siRNA was used to knock down *Bcl*‐*2* in H9c2 cells (Figure 3E). As shown in Figure 3F, the protective effects of Dex were attenuated by silencing *Bcl*‐*2*. Furthermore, the inhibitory effects of Dex on the activation of caspase‐3 were abrogated by the downregulation of *Bcl*‐*2* (Figure 3G,H). Therefore, Dex exerted its protective effects via the inhibition of the intrinsic pathway. Because ER stress is known to be essential for H/R‐induced apoptosis,^10^ the effects of Dex on ER stress were examined. The H/R treatment induced the upregulation of GRP78 and CHOP (Figure 3I). Pretreatment with Dex inhibited the upregulation of GRP78 and CHOP caused by H/R exposure in H9c2 cells (Figure 3I). To study the function of the ER stress pathway in the protective effects of Dex, thapsigargin, an ER stress activator, was applied. As shown in Figure 3J, thapsigargin (1 μM) treatment successfully reversed the effects of Dex on ER stress. It was observed that the protective effects of Dex were diminished by thapsigargin in H9c2 cells (Figure 3K). Furthermore, the inhibitory effects of Dex on the activation of caspase‐3 were abrogated by thapsigargin in H9c2 cells (Figure 3L,M). Altogether, these data suggested that Dex exerts its cardioprotective effects against H/R injury by inhibiting apoptosis and ER stress in H9c2 cells.

### 3.4 Dex activates JAK2/STAT3 pathway

**FIGURE 4:**
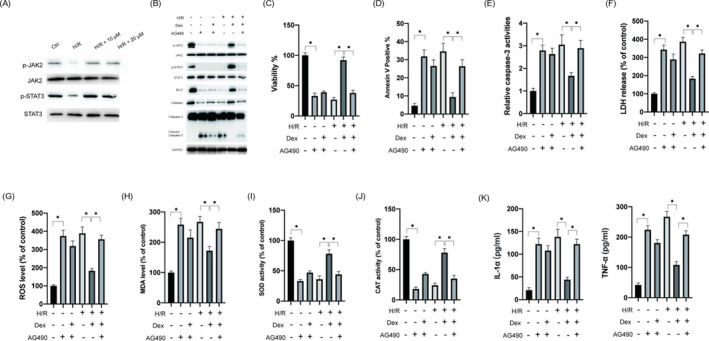
Dex treatment led to the activation of JAK2/STAT3 in H9c2 cells. (A) H9c2 cells were pretreated with indicated doses of Dex for 2 h, following which cells were exposed to H/R conditions, total cellular lysates were subjected to Western blotting. (B) H9c2 cells were pretreated with Dex (20 μM) with or without AG490 (50 μM) for 2 h, following which cells were exposed to H/R conditions and cellular lysates were subjected to Western blotting. (C) Cell viabilities were measured. (D) Apoptosis was measured. (E) Relative caspase‐3 activities were measured. (F) The release of LDH was measured. (G) ROS levels were measured. (H) MDA levels were measured. (I) SOD activities were measured. (J) CAT activities were measured. (K) Levels of IL‐1α (left) and TNF‐α (right) were measured. Data are presented as mean ± SD, *p < 0.05; **p < 0.01

We next examined the status of the JAK2/STAT3 signaling pathway as it has been shown to play an essential role in ER stress.^11^ As shown in Figure 4A, H/R inhibited the phosphorylation of JAK2 and STAT3, whereas Dex treatment led to the activation of the JAK2/STAT3 pathway. The STAT3 inhibitor AG490 (10 μM) markedly inhibited the JAK2/STAT3 pathway in the presence of Dex (Figure 4B). Furthermore, we observed that AG490 treatment abrogated the protective effects of Dex against H/R injury (Figure 4C,D). The treatment of AG490 rescued the activation of caspase‐3 in the presence of Dex under H/R injury (Figure 4B,E). Furthermore, the inhibitory effects of Dex on the levels of LDH, ROS, and MDA were diminished following AG490 treatment (Figure 4F–H). The effects of Dex on the activities of SOD and CAT were also mitigated by AG490 treatment (Figure 4I,J). In addition, the administration of AG490 abrogated the inhibition of Dex‐induced IL‐1α and TNF‐α in H9c2 cells (Figure 4K). AG490 inhibited the expression of catalase in the presence of Dex and H/R in H9c2 cells (Figure 4B). These data suggested that Dex led to the activation of the JAK2/STAT3 pathway under the H/R condition.

### 3.5 Activation of the JAK2/STAT3 pathway is responsible for catalase upregulation

**FIGURE 5:**
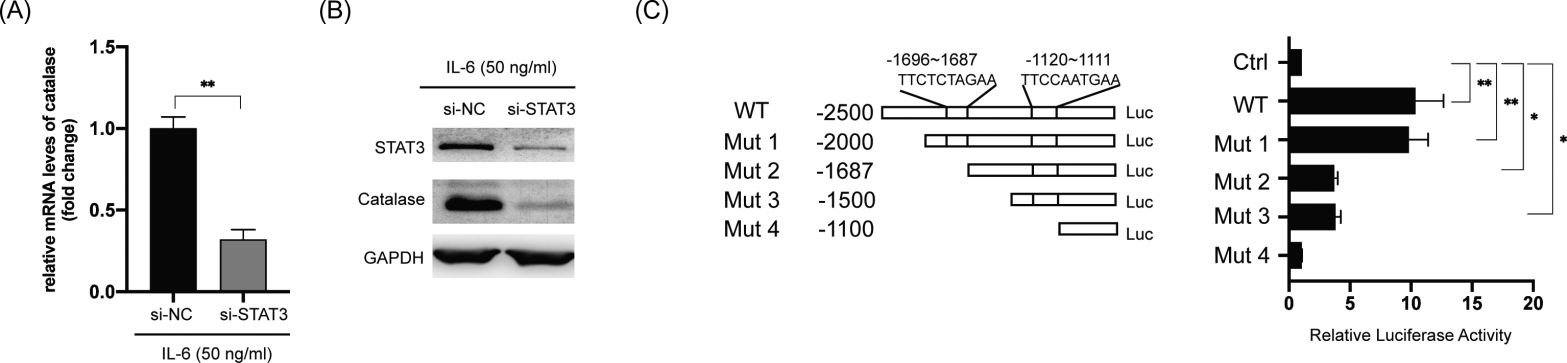
STAT3 is responsible for the transcription of catalase. (A) H9c2 cells were transfected with si‐NC or si‐STAT3 for 4 h, following which cells were treated with IL‐6 for another 6 h, and mRNA levels of catalase were measured. (B) H9c2 cells were transfected with si‐NC or si‐STAT3 for 4 h, following which cells were treated with IL‐6 (50 ng/ml) for another 12 h. The protein levels of STAT3 and catalase were measured. (C) Schematic structures of the catalase promoter constructs used to measure the luciferase activity (left) are shown. H9c2 cells were transfected with the reporter constructs for 4 h and treated with IL‐6 (50 ng/ml) for another 6 h, following which luciferase activities were measured. Data are presented as mean ± SD, *p < 0.05; **p < 0.01

Based on the above findings, we hypothesized a correlation between STAT3 and catalase. To test this, si‐STAT3 was transfected into H9c2 cells; silencing of *STAT3* repressed both the mRNA and protein levels of catalase following treatment with IL‐6 (50 ng/ml) (Figure 5A,B). To examine whether STAT3 directly induced the expression of catalase, potential STAT3‐binding sites in the regulatory regions of the *catalase* gene were searched. We used the online bioinformatic tools PROMO (http://alggen.lsi.upc.es/cgi‐bin/promo_v3/promo/promoinit.cgi?dirDB=TF_8.3) and JASPAR (http://jaspar.genereg.net) and found two putative STAT3‐binding sites located on the upstream of catalase gene (Figure 5C, left). Dual‐luciferase activity assay showed that STAT3 could bind to the promoter region of the catalase gene (Figure 5C, right). Altogether, these data suggested that Dex‐induced upregulation of catalase was dependent on the activation of the JAK2/STAT3/catalase axis.

## 4 DISCUSSION

We found that Dex protected H9c2 cells from H/R‐induced injury as evidenced by enhanced cell viability, and decreased oxidative stress, inflammatory response, and apoptosis. Furthermore, the protective effects of Dex were correlated with the alleviation of ER stress and activation of the JAK2/STAT3/catalase axis.

Increasing evidence has suggested that Dex, a sedation drug, possesses anti‐inflammatory, anti‐apoptotic, and antioxidative stress effects.^12^ Although Dex has been reported to exert cardioprotective effects, the underlying mechanisms have remained elusive. H/R condition has widely been recognized as an in vitro model to mimic myocardial I/R injury.^13^ Thus, myocardial I/R injury was established in H9c2 cells to investigate the protective effects of Dex. Our results showed that Dex significantly improved the cell viability of H9c2 cells under H/R conditions. These results are in line with those of previous studies that also found that Dex protected H9c2 cells from H/R injury.^14^, ^15^

Apoptosis plays an essential role in myocardial I/R injury.^2^ There are mainly two apoptotic pathways, namely the extrinsic and intrinsic pathways.^16^ The intrinsic pathway is regulated by the Bcl‐2 family proteins. We found that H/R treatment led to the activation of the intrinsic apoptosis pathway, a finding in line with a previous study.^17^ To confirm that the inhibition of intrinsic apoptosis is critical for the protective effects of Dex, Bcl‐2 was silenced; the protective effects of Dex were diminished following the knockdown of Bcl‐2. These findings are following those of a previous study that also reported that the overexpression of Bcl‐2 inhibited H/R‐induced injury.^17^ Hence, the cardioprotective effects of Dex were closely correlated with H/R‐induced inhibition of intrinsic apoptosis.

ER stress has been implicated in the progression of myocardial I/R injury and ischemic myocardial cells apoptosis.^18^ Various agents are known to exert protective effects against I/R‐induced injury via the alleviation of ER stress. Therefore, targeting ER stress could be a potential strategy for the treatment of MI. To this end, we found that treatment with Dex inhibited ER stress markers, namely GRP78 and CHOP under H/R conditions. In addition, the administration of thapsigargin successfully abrogated the protective effects of Dex. These findings are in line with those of a recent study that also reported that Dex released ER stress in H/R‐treated H9c2 cells.^19^

It is well‐documented that oxidative stress is closely correlated with I/R injury.^18^ We found that Dex pretreatment alleviated oxidative stress in H/R‐treated H9c2 cells. Our findings are following those of previous studies that suggest that Dex ameliorates oxidative stress in different cells.^14^, ^20^ However, little is known about the mechanisms underlying the antioxidative effects of Dex. We demonstrated that Dex treatment upregulated catalase and inhibited catalase, thereby partially inhibiting the protective effects of Dex. The silencing of catalase cannot completely inhibit the protective effects of Dex, which could be attributed to the efficiency of knockdown and/or other antioxidative enzymes that may compensate for the loss of catalase. Altogether, these data suggest that Dex exerts cardioprotective effects against I/R injury via the inhibition of oxidative stress.

The JAK2/STAT3 signaling pathway is involved in several physiological activities including MI.^21^ Numerous studies suggest that the activation of the JAK2/STAT3 signaling pathway diminishes the myocardial I/R injury.^22^ We found that pretreatment with Dex activated the JAK2/STAT3 pathway under H/R conditions. Furthermore, the protective effects of Dex were reversed by the administration of AG490, and both AG490 treatment and STAT3 silencing decreased the levels of catalase. An investigation of the mechanism showed that STAT3 promoted the expression of catalase. This finding is in line with an earlier study that also found that STAT3 promoted the expression of catalase.^23^ Interestingly, it was also reported that catalase inhibited the activation of STAT3.^24^ Therefore, a feedback loop may exist between STAT3 and catalase, and further investigation is required to test it. These data suggest that Dex exerts cardioprotective effects at least in part through the JAK2/STAT3/Catalase axis.

## 5 CONCLUSION

In summary, our findings demonstrated that Dex protected H9c2 cells against H/R injury by inhibiting the intrinsic apoptosis pathway and ER stress, and activating the JAK2/STAT3/Catalase axis. However, we only evaluated the cardioprotective effects of Dex; in vitro and in vivo investigations will be considered in the coming studies.

## CONFLICT OF INTEREST

All authors declare that they have no conflict of interests.

## Data Availability

The data that support the findings of this study are available from the corresponding author upon reasonable request.
